# Overcoming intratumoural heterogeneity for reproducible molecular risk stratification: a case study in advanced kidney cancer

**DOI:** 10.1186/s12916-017-0874-9

**Published:** 2017-06-26

**Authors:** Alexander L. R. Lubbock, Grant D. Stewart, Fiach C. O’Mahony, Alexander Laird, Peter Mullen, Marie O’Donnell, Thomas Powles, David J. Harrison, Ian M. Overton

**Affiliations:** 1 MRC Institute of Genetics and Molecular Medicine, University of Edinburgh, Edinburgh, EH4 2XU UK; 2 Scottish Collaboration On Translational Research into Renal Cell Cancer (SCOTRRCC), Scotland, UK; 3 School of Medicine, University of St Andrews, St Andrews, Fife, KY16 9TF UK; 4 Department of Pathology, Western General Hospital, Edinburgh, EH4 2XU UK; 5 Barts Cancer Institute, Experimental Cancer Medicine Centre, Queen Mary University of London, London, EC1M 6BQ UK; 6 Usher Institute of Population Health Sciences and Informatics, University of Edinburgh, Edinburgh, EH16 4UX UK; 7 Present Address: Vanderbilt University School of Medicine, Vanderbilt University, Nashville, Tennessee USA; 8 Present Address: Academic Urology Group, University of Cambridge, Box 43, Addenbrooke’s Hospital, Cambridge Biomedical Campus, Hill’s Road, Cambridge, CB2 0QQ UK

**Keywords:** Cancer, Tumour heterogeneity, Prognostic markers, Renal cell carcinoma, Tumour biomarkers

## Abstract

**Background:**

Metastatic clear cell renal cell cancer (mccRCC) portends a poor prognosis and urgently requires better clinical tools for prognostication as well as for prediction of response to treatment. Considerable investment in molecular risk stratification has sought to overcome the performance ceiling encountered by methods restricted to traditional clinical parameters. However, replication of results has proven challenging, and intratumoural heterogeneity (ITH) may confound attempts at tissue-based stratification.

**Methods:**

We investigated the influence of confounding ITH on the performance of a novel molecular prognostic model, enabled by pathologist-guided multiregion sampling (*n* = 183) of geographically separated mccRCC cohorts from the SuMR trial (development, *n* = 22) and the SCOTRRCC study (validation, *n* = 22). Tumour protein levels quantified by reverse phase protein array (RPPA) were investigated alongside clinical variables. Regularised wrapper selection identified features for Cox multivariate analysis with overall survival as the primary endpoint.

**Results:**

The optimal subset of variables in the final stratification model consisted of N-cadherin, EPCAM, Age, mTOR (NEAT). Risk groups from NEAT had a markedly different prognosis in the validation cohort (log-rank *p* = 7.62 × 10^−7^; hazard ratio (HR) 37.9, 95% confidence interval 4.1–353.8) and 2-year survival rates (accuracy = 82%, Matthews correlation coefficient = 0.62). Comparisons with established clinico-pathological scores suggest favourable performance for NEAT (Net reclassification improvement 7.1% vs International Metastatic Database Consortium score, 25.4% vs Memorial Sloan Kettering Cancer Center score). Limitations include the relatively small cohorts and associated wide confidence intervals on predictive performance. Our multiregion sampling approach enabled investigation of NEAT validation when limiting the number of samples analysed per tumour, which significantly degraded performance. Indeed, sample selection could change risk group assignment for 64% of patients, and prognostication with one sample per patient performed only slightly better than random expectation (median logHR = 0.109). Low grade tissue was associated with 3.5-fold greater variation in predicted risk than high grade (*p* = 0.044).

**Conclusions:**

This case study in mccRCC quantitatively demonstrates the critical importance of tumour sampling for the success of molecular biomarker studies research where ITH is a factor. The NEAT model shows promise for mccRCC prognostication and warrants follow-up in larger cohorts. Our work evidences actionable parameters to guide sample collection (tumour coverage, size, grade) to inform the development of reproducible molecular risk stratification methods.

**Electronic supplementary material:**

The online version of this article (doi:10.1186/s12916-017-0874-9) contains supplementary material, which is available to authorized users.

## Background

There is a great unmet need for better treatment and diagnosis of kidney cancer, which remains the most lethal of all genitourinary malignancies. Five-year survival in renal cell cancer (RCC) is approximately 40% overall, 10% in metastatic disease [1, 2]. Clear cell RCC (ccRCC) represents about 80% of cases, and around one-third of patients present with metastasis. Current risk stratification of advanced ccRCC uses clinico-pathological scoring systems, for example, the International Metastatic Database Consortium (IMDC) [3] and Memorial Sloan Kettering Cancer Center (MSKCC) [4] scores. Molecular markers promise to overcome the performance plateau encountered by clinico-pathological variables; however, success rates have historically been low [5–8].

Sunitinib is a first-line treatment for metastatic ccRCC (mccRCC), doubling median progression-free survival compared with older immunotherapies such as IL-2 and interferon-α [9, 10]. Sunitinib targets tumour, endothelial cells and pericytes, where the mechanism of action includes competitive inhibition of multiple receptor tyrosine kinases (RTKs) [11, 12]. Up to 70% of patients treated with sunitinib show little or no tumour response [10], although they may derive a survival benefit, despite incurring significant toxicity. Improved algorithms are critically needed to guide treatment decisions for current and emerging modalities [6, 7, 13].

Advances in prediction of treatment response and prognostication may be severely hindered by intratumoural heterogeneity (ITH) [14–16]. Indeed, percutaneous biopsy of mccRCC is a poor guide for pathological assessment of prognostic features [17]. Development of tumour sampling approaches to capture ITH is key for discovery and validation of candidate molecular risk stratification algorithms [6, 7, 13, 15]. We studied protein expression ITH in the context of mccRCC risk stratification, controlling for clinical variables, and developed a novel prognostic model (NEAT, for N-cadherin, EPCAM, Age, mTOR) that compares well with established clinico-pathological scores. The variables selected in NEAT inform mccRCC biology and suggest sunitinib action directly on tumour growth signalling. We quantitatively show a dramatic effect of tumour sampling on NEAT performance in a validation cohort receiving current standard treatment and demonstrate parameters pertinent to the development of molecular diagnostic tools for cancer medicine. We present recommendations that guide tumour sample selection for biomarker research in order to overcome variability in the presence of ITH. Indeed, sampling protocols may determine the success or failure of attempts to validate molecular biomarkers where ITH is a factor.

## Methods

### Cohorts and tissue samples

This study examined two geographically separated cohorts of mccRCC patients with multiregion tumour sampling (Table 1). Excluding necrotic tissue, 108 and 75 fresh-frozen samples respectively were analysed from development and validation cohorts. The development cohort was drawn from the SuMR phase II clinical trial of upfront sunitinib (NCT01024205, *n* = 22, London [18]). The validation cohort were cytoreductive nephrectomy patients from the SCOTRRCC study and received standard of care treatment (validation, *n* = 22, Scotland [1, 19]). The development cohort received three cycles of sunitinib 50 mg (4 weeks on, 2 weeks off) prior to nephrectomy; following nephrectomy, the validation cohort received either sunitinib (*n* = 8), similar targeted agents (*n* = 3) or no drug (*n* = 11). These cohorts were enriched for patients with a poor or intermediate prognosis, in line with the SuMR trial selection criteria [18]. Median follow-up time, defined as time of entry to death or last contact, was 22.0, 12.3 months respectively for the development, validation cohorts. Univariate Cox regression for mTOR and overall survival analysed an overlapping cohort (*n* = 45) which included an additional patient [20]. Comparisons of cohort characteristics used Mann–Whitney, Fisher or binomial tests as appropriate; *p* values were two-tailed and corrected for multiple hypothesis testing [21]. Net reclassification improvement (NRI) confidence intervals were calculated using bootstrapping [22, 23].

**Table 1:** Clinical characteristics of cohorts studied

Cohort	Total	Development (SuMR)	Validation (SCOTRRCC)
Number of patients	44	22	22
Age, median (range)^a^	66.5 (38.2–79.3)	64.5 (44–78)	67.5 (38.2–79.3)
Overall survival (months):^b^			
Median	16.2	23.5	12.3
Interquartile range	9.1–25.9	14.8–29.8	7.6–18.8
Number censored	22	10	12
Modes (bimodal model)	10.6, 27.3	10.2, 27.4	11.6, 27.5
Number of samples per tumour^a^ median (range)	4 (1–10)	4 (1–10)	4 (2–8)
Male gender^b^	29	15	14
Fuhrman grade:^b^			
4	9	1	8
3	22	12	10
2	13	9	4
Stage:^b^			
T4	4	4	0
T3	33	13	20
T2	6	4	2
T1	1	1	0
Performance status:^c^			
KPS >80 (unavailable)	34 (1)	13 (0)	21 (1)
Anaemia^b^^,+^ (unavailable)	22 (2)	12 (0)	10 (2)
Raised calcium^b^ (unavailable)	8 (7)	5 (0)	3 (7)
Raised LDH^c^ (unavailable)	10 (15)	5 (0)	5 (15)
Neutrophil count >70% upper limit of normal^b^ (unavailable)	9 (2)	2 (0)	7 (2)
Platelet count >400^b^ (unavailable)	12 (2)	6 (0)	6 (2)
*VHL* mutation^b^	31	14	17
Renal response at surgery:			
Partial response	–	2	–
Stable disease	–	20	–
Number of metastatic sites:^b^			
1	19	6	13
2	18	11	7
3	7	5	2
IMDC class:^b^			
Intermediate	26	12	14
Poor	16	10	6
Unavailable	2	0	2
MSKCC class:^b^			
Favourable/intermediate:	26	13	13
Poor	12	9	3
Unavailable	6	0	6

^+^Hb <130 (M), <110 (F); ^a^ *p <* 0.05; ^b^ *p* > 0.05; ^c^ *p* < 10^-4^

### Multiregion tumour sampling

Details of multiregion tissue mapping and sample preparation are given in [24]. Briefly, samples taken forward for reverse phase protein array (RPPA) analysis were spatially separated and selected to represent morphological diversity across the tumour. Fresh-frozen tumours were divided into spatially mapped 1 cm^3^ pieces; cryostat sections of each piece were examined to confirm ccRCC status and for morphological classification. Up to four samples per morphologically distinct region in each tumour were selected for protein extraction; each of these samples reflected *circa* 50–75 mm^3^ of tissue.

### Intratumoural protein expression variance in sunitinib-exposed and sunitinib-naïve cancers

Fifty-five protein targets were investigated by RPPA, selected according to prior knowledge and validated antibody availability [20]. Each tumour sample analysed by RPPA reflected 50–75 mg of lysed tissue taken from a 1 cm^3^ spatially mapped region [24]. Protein extraction, RPPA slide spotting, immunofluorescence data acquisition, data processing and identification of four markers that had increased variance associated with sunitinib treatment (*p* < 0.05) were described previously [20, 25]. Briefly, 1 mg/ml lysates were spotted onto nitrocellulose slides using a robotic spotter, and immunofluorescence imaging was performed with an Odyssey scanner (Li-Cor Biosciences, Lincoln, NB, USA). Image processing and logistic curve fitting to the RPPA dilution series employed MicroVigene software (VigeneTech, Carlisle, MA, USA). Protein variance per tumour was estimated using batch-corrected, normalised RPPA expression values from multiregion sampling, comparing the ratio of mean-squared errors between sunitinib-exposed and sunitinib-naïve cohorts per protein marker in an analysis of variance (ANOVA) framework. Statistical significance of variance differences was assessed using the *F* test only when relevant assumptions held, assessed by the Lillefors and Fligner-Kileen tests [20]. Ranking by the protein expression variance log-ratio between sunitinib-exposed and sunitinib-naïve tumours identified a further two proteins of potential interest where variance was greater than at least one of the four significant markers; these proteins did not meet *F*-test assumptions and so were not assessed in our previous work using the ANOVA framework. Therefore, six proteins (CA9, N-cadherin (CDH2), EPCAM, mTOR (MTOR), MLH1, BCL2) were candidate molecular variables input into feature selection (described in the following section). The antibodies used for these candidate variables are listed in (supplementary) Table S1 of Additional file 1.

### Selection of variables and multivariate modelling

Variables were selected for Cox proportional hazards regression to overall survival on the development cohort using wrapper feature selection with backward elimination regularised by Bayesian information criterion (BIC) [26, 27]. Backward elimination iteratively removed a single feature (i.e. protein expression or a clinical parameter) at each step, selecting for the greatest improvement in BIC value. BIC regularisation seeks to balance the model complexity (number of parameters, including candidate features) against the model likelihood (fit to the data); therefore, this approach removes features with the smallest contribution to model likelihood while penalising redundancy. The selection procedure terminated with a final model when removing any single feature did not improve BIC. The 'coxph' and 'stepAIC' functions were used respectively from the 'survival' and 'MASS' R libraries (with model complexity penalty specified for BIC) [28].

### Comparison with established clinico-pathological scores

IMDC and MSKCC scores were calculated according to the relevant clinical parameters [3, 4]. Sufficient data were available to calculate the IMDC score for 20/22 patients in the validation cohort, all of whom fell into the 'intermediate' or 'poor' categories. MSKCC score was used to group patients into (1) favourable/intermediate and (2) poor prognosis; sufficient data were available to classify 14/22 patients. A further two patients were on the borderline of intermediate or poor prognosis with MSKCC parameters due to missing data, but had short survival times and were assigned to the poor prognosis group. Therefore, two ambiguous values were resolved in favour of the MSKCC score performance, making comparison with NEAT more stringent; hence 16/22 patients were assigned MSKCC scores. All patients in the development cohort had sufficient data for IMDC and MSKCC scoring. The reported hazard ratio (HR) for NEAT reflects stratification into either better or worse than average risk groups (i.e. classification threshold of logHR = 0); this threshold was predetermined and not derived from exploratory data analysis. HR reported for IMDC, MSKCC follows the groupings described above.

### Investigating stratification performance with reduced number of samples per tumour

In order to evaluate tumour sampling effects on NEAT performance, a subsampling procedure produced datasets taking a maximum number of tumour samples (MNTS) of 1, 2 or 3 per tumour (and thus per patient). This approach employed Sobol sampling [29]; see supplementary methods in Additional file 1 for further details. The selected tumour samples were used to calculate median protein expression per patient as input for the NEAT algorithm. Patient age was unchanged. The HR and log-rank *p* value for stratification into 'high' and 'low' risk groups defined by NEAT logHR = 0 were calculated. This analysis was performed on 10^6^ datasets per MNTS examined, where each dataset represented a unique combination of samples across all patients in the validation cohort. Therefore, every patient was represented in each of the 10^6^ datasets; thus, 10^6^ NEAT HR and log-rank *p* values were generated for each MNTS, representing predictive performance distributions across the different tumour sample combinations.

## Results

### Cohort characteristics

**Fig. 1:**
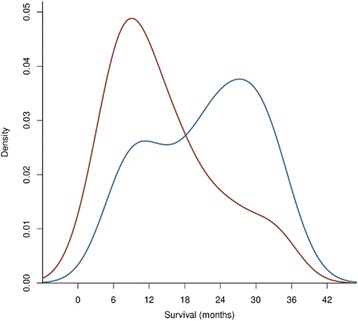
Overall survival distributions for the development (SuMR) and validation (SCOTRRCC) cohorts. Kernel density plots are shown for all survival data, including censored data. The above distributions indicate bimodality for both cohorts studied, with similar mode positions around 11 and 27 months. These survival modes align with survival subgroups reported in pivotal studies [3, 4, 30]. The development cohort (*blue*) had the greatest proportion of patients in the mode centred around 27 months, reaching a density value of 0.037. The majority of patients in the validation cohort (*red*) are in the survival mode around 11 months (reaching a density value of 0.049), partly due to greater censoring in this cohort

The two mccRCC cohorts were similar across many characteristics (Table 1), although statistically significant differences were identified for Karnofsky performance status, elevated lactate dehydrogenase and age. Clustering analysis of overall survival (OS) using regularised Gaussian mixture modelling for unsupervised cardinality selection identified two modes (clusters) in the combined cohorts (*n* = 44, Fig. 1). The longer survival cluster had a median OS (mOS) of 27.3 months, matching the favourable or intermediate prognosis subgroups defined in pivotal studies. For example, the favourable subgroup reported for the MSKCC score had mOS of 30 months [4], mOS for the IMDC score intermediate subgroup was 27 months [3] and a further independent study reported mOS of 26 months for the favourable subgroup [30]. The shorter survival cluster had mOS of 10.6 months, which is similar to reported mOS values across poor and intermediate prognosis subgroups in the preceding studies [3, 4, 30]. Greater representation of the shorter survival cluster in the validation cohort was partly due to censoring and also arose from the drug response selection criterion for the development cohort [18]. However, survival times for the validation and development cohorts were not significantly different. Therefore, the population studied (*n* = 44) has a bimodal OS distribution that aligns with that of subgroups identified in larger mccRCC cohorts [3, 4, 30].

### The NEAT algorithm for risk stratification of patients with metastatic renal cancer

**Fig. 2:**
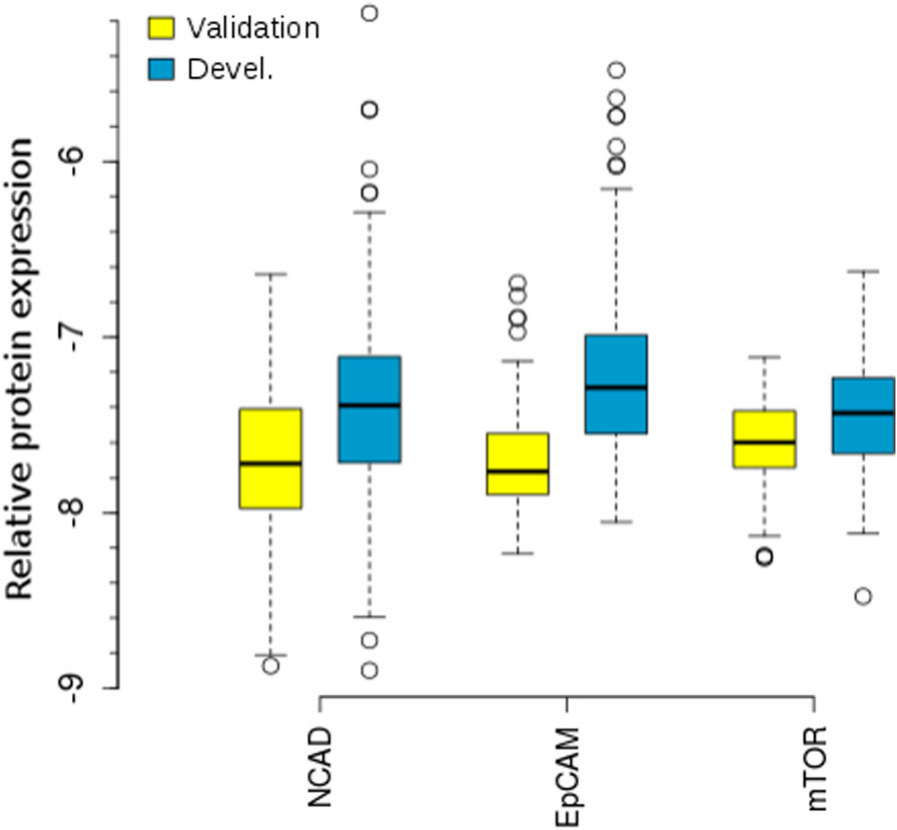
Expression values for NEAT molecular features. Protein concentration values determined by RPPA for validation (*yellow*) and development (*blue*) cohorts are shown for all samples (log2), including multiple datapoints per tumour. Therefore, a single tumour may contribute datapoints across the full range of expression values shown in each box plot. Relative expression values increase from the bottom (−9) to the top (−5.2) of the *y*-axis. The distributions are overlapping, with a shift towards higher expression in the development cohort

A machine learning approach using regularised wrapper selection [27] with Cox multivariate analysis [26] on the development cohort identified a novel model for mccRCC patient risk stratification by overall survival. We hypothesised that proteins with increased intratumoural variance following therapy may function as markers of resistance or aggressiveness and so enable prognostication. Indeed, factors underlying changes in tumour composition with treatment include clonal selection and proteomic diversity across isogenic cell populations [16, 31, 32]. Twelve variables were examined, including six key clinical parameters (grade, gender, age, neutrophils, haemoglobin, IMDC score [3]) and values for six proteins where intratumoural variance was greater in sunitinib-exposed mccRCC. Prognostic variables automatically identified by machine learning were N-cadherin, EPCAM, Age and mTOR (NEAT), controlling for the above clinical parameters. Protein expression values for these markers in the development and validation cohorts are shown in Fig. 2. The resulting multivariate Cox proportional hazards model for the development cohort had likelihood ratio test *p* = 1.18 × 10^−4^, and all selected variables were individually significant in the multivariate model (Table 2).

**Table 2:** Multivariate Cox proportional hazards model for overall survival, fitted on the development cohort

Feature	HR (95% confidence interval)	*p*
mTOR	1.04 × 10^–8^ (6.09 × 10^–4^ – 1.71 × 10^–13^)	0.001
EPCAM	44.7 (845.6–2.36)	0.011
N-Cadherin	7.53 × 10^3^ (2.76 × 10^6^ – 20.71)	0.003
Age	1.14 (1.27–1.02)	0.018

The interesting positive relationship of mTOR with survival was followed up in an overlapping cohort and was significant in univariate Cox regression (*p* = 0*.*034). The proportional hazards assumption was not invalidated (Grambsch-Therneau test [33], (supplementary) Table S2 of Additional file 1). The HR was calculated from relative protein expression values and age in years at diagnosis as follows:

Hazard ratio = exp(8.927 N-cadherin + 3.800 EPCAM + 0.129 Age - 18.385 mTOR)

**Fig. 3:**
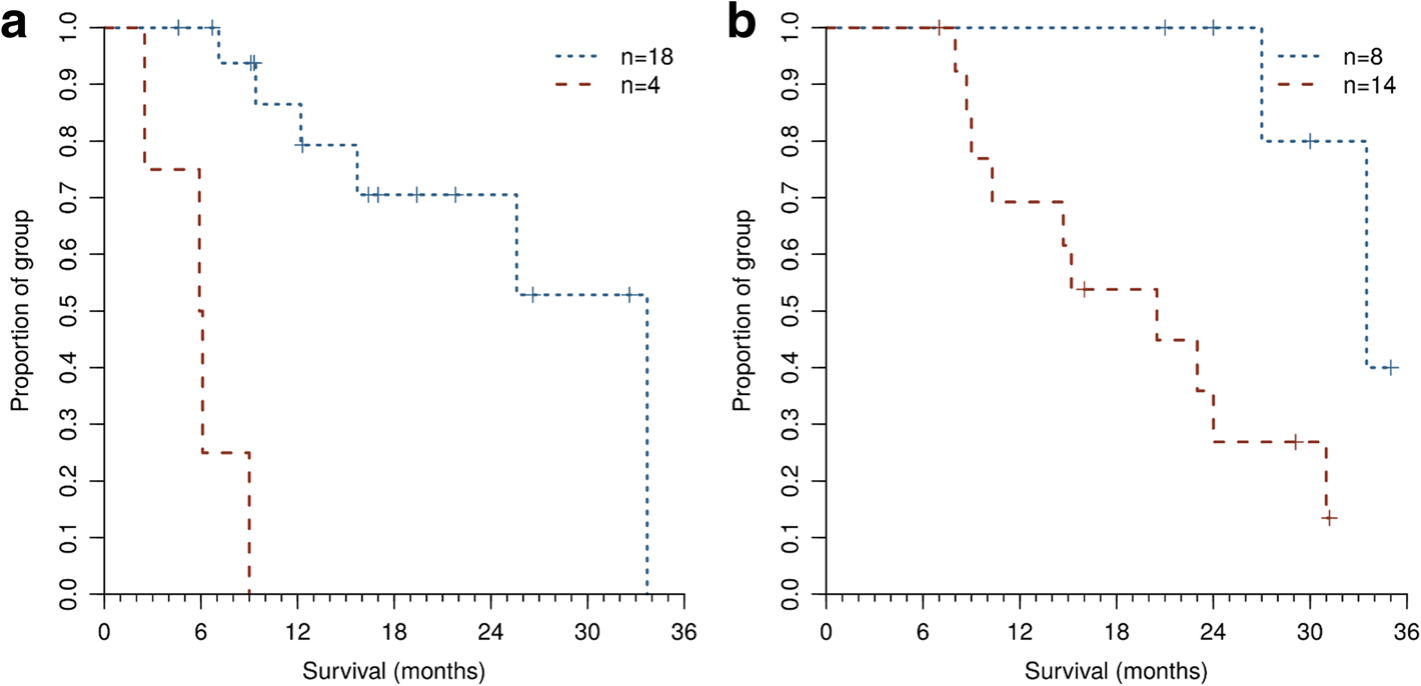
Kaplan-Meier curves for overall survival risk stratification by N-Cadherin, EPCAM, Age and mTOR (*NEAT*). **a** Validation cohort. The high risk (*n* = 4, *dashed line*) and low risk (*n* = 18, *dotted line*) groups identified by NEAT have markedly different prognoses (log-rank *p* = 7.62 × 10^−7^) with respective 2-year survival rates of 0% and 78% (precision = 100%, recall = 50%, specificity = 100%, accuracy = 82%, Matthews correlation coefficient = 0.62). Data analysed were independent of wrapper feature selection and of fitting model coefficients. **b** Development cohort. The identified features and model coefficients were learned on the data shown, which therefore does not provide an independent test. High risk (*n* = 14, *dashed line*) and low risk (*n* = 8, *dotted line*) groups are clearly separated (log-rank *p* = 0.00553), with respective 2-year survival rates of 43% and 100%. (precision = 57%, recall = 100%, specificity = 57%, accuracy = 73%, Matthews correlation coefficient = 0.57)

NEAT performed well on the geographically separated validation and development cohorts (Fig. 3). This work reflects evidence level IB [34], where development used prospective clinical trial data and validation was performed with patients who received current standard therapy. Concordance index (*C* index) [35] values for the NEAT, IMDC and MSKCC score risk groups in the validation cohort were respectively 0.77 (95% CI 0.66–0.88), 0.76 (95% CI 0.60–0.92) and 0.64 (95% CI 0.54–0.75). Net reclassification improvement [22] values for NEAT on the validation cohort were 7.1% vs IMDC (95% CI −24.8%, 39.0%) and 25.4% vs MSKCC score (95% CI −25.7%, 76.5%), shown in Table 3.

**Table 3:** Performance characteristics of NEAT and clinico-pathological scores

Cohort	Percentage NRI (95% CI)		HR (95% CI)		
	NEAT vs IMDC	NEAT vs MSKCC^+^	NEAT	IMDC	MSKCC
Validation	7.1^a^ (–24.8, 39.0)	25.4^b^ (–25.7, 76.5)	37.89 (4.1, 353.8)	7.317^a^ (1.54, 34.69)	14.39^b^ (1.24, 165.7)
Development	25.0 (–34.8, 84.8)	23.3 (–28.3, 74.9)	10.649 (1.35, 84.08)	1.378 (0.44, 4.36)	3.577 (1.00, 12.85)

*NRI* net reclassification improvement, *HR* Hazard ratio, *NEAT* N-cadherin EPCAM Age mTOR multivariate model, *IMDC* International Metastatic Database Consortium, *MSKCC* Memorial Sloan Kettering Cancer Center

Values reflect assignment of either high risk or low risk status (methods)

^a^ *n* = 20 due to data availability

^b^ *n* = 16 due to data availability

### Tumour sampling is a critical limiting factor for validation of molecular stratification approaches

**Fig. 4:**
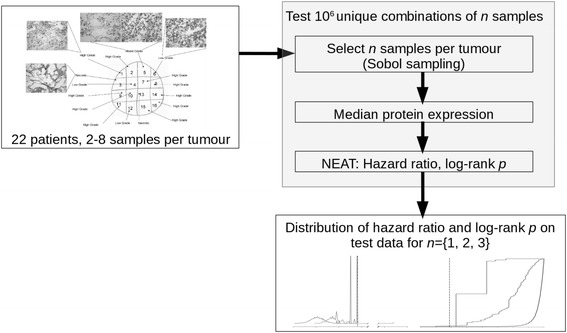
Overall approach for investigation of the effect of subsampling on NEAT predictive performance. A total of 10^6^ combinations of *n* = {1,2,3} samples per tumour were analysed across the 22 patients in the validation cohort where multiregion sampling encompassed identified morphological intratumoural heterogeneity (*top left*). A median of four samples was taken per tumour. The distributions of logHR and log-rank *p* values across the 10^6^ samples taken for each value of *n* (*bottom right*) are given at readable size in Fig. 5

**Fig. 5:**
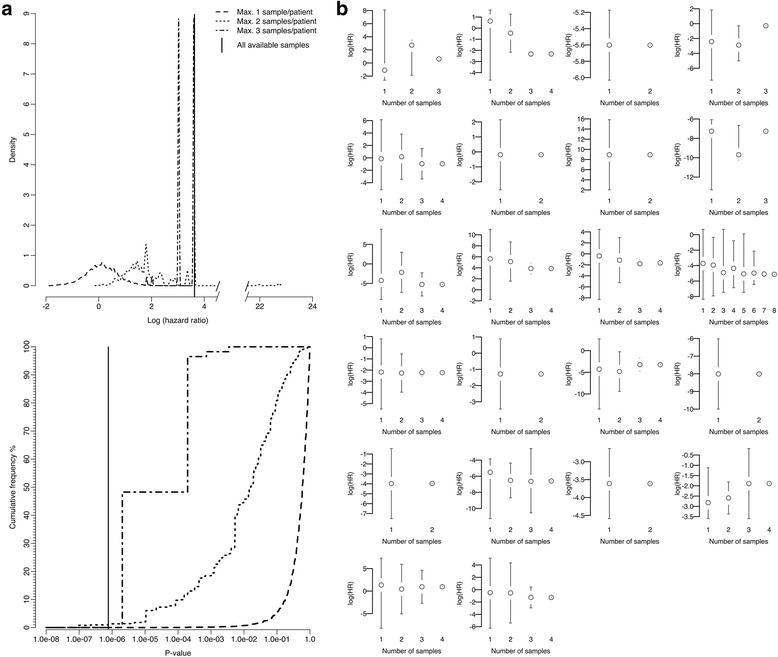
Stratification of the validation cohort critically depends upon tumour sampling. **a** Values of NEAT logHR (*top*) and *p* values (log-rank test, *bottom*) are shown for subsampled datasets generated by taking a maximum of one (*dashed line*), two (*dotted line*) or three (*dot-dash line*) samples per tumour. The *vertical line* in each graph indicates NEAT performance using all available samples. Stratification performance improves significantly as the number of samples taken increases. **b** Variation in per-patient NEAT HR driven by tumour sampling. Each plot corresponds to a patient and shows the distribution of logHR from NEAT across the available tumour samples. *Vertical bars* indicate logHR range for every possible combination of the specified number of samples. Therefore, logHR calculated using all samples is shown on the *right* of each plot as a *single point*. For many patients (14/22, 64%) the logHR distribution encompasses the classification threshold (logHR = 0); hence risk group assignment is critically influenced by the tumour sample(s) analysed

The overall approach to investigate the effects of tumour sampling on predictive performance is summarised in Fig. 4. Three distributions of NEAT hazard ratio and log-rank *p* value were generated to reflect sampling 1, 2 or 3 regions per tumour in the validation cohort; these distributions capture NEAT performance for different sample combinations taken across tumours and patients. For example, consider three patients, each with RPPA data from four different tumour samples. If a single sample is taken from each patient for NEAT analysis, there would be 4^3^ (i.e. 64) unique combinations of tumour samples across the three patients. Validation power rose significantly at each increase in the number of tumour samples taken per patient, and the full dataset with a median of four spatially separated samples per tumour appeared adequate, conferring good predictive power. NEAT overall performance on the validation cohort was poor when limited to a single tumour sample per patient, and was significantly impaired with two samples per patient (Fig. 5a). In the single sample regime, stratification into good and poor prognosis groups was only just better than random expectation (median logHR = 0.109, binomial *p* < 10^−322^); strong statistical significance is due to the large datasets studied. Taking two samples per tumour gave improved stratification performance over a single sample (median logHR = 1.614, Mann–Whitney *p* < 10^−324^), and substantial further improvement was found when taking three samples (median logHR = 3.030, Mann–Whitney *p* < 10^−324^). Application of NEAT to different subsets of tumour samples per individual patient changed risk group assignment for 64% of the validation cohort (Fig. 5b). Interestingly, the median variance in per-patient HR was 3.5-fold greater in low grade samples than high grade samples (Mann–Whitney *p* = 0.044). In order to further investigate the independent prognostic power of individual tumour regions, we compared prediction using expression values averaged across all available samples for each individual against the best possible results obtained using only one sample per tumour. Validation using all of the available samples per tumour outperformed even the most predictive single sample taken (*p* < 10^−6^).

## Discussion

This study examines the effect of sampling on the performance of a novel molecular prognostic approach, NEAT, using protein measurements from 183 regions across 44 mccRCC tumours. The unique development cohort from the SuMR trial allowed for selection of proteins that had increased intratumoural expression variance with treatment; we hypothesised that these proteins may be markers of aggressiveness and therefore useful in prognostication. Although the cohorts are relatively small, NEAT gave statistically robust stratification of the independent validation cohort by overall survival (Fig. 3a). The trend for favourable NEAT performance relative to the IMDC, MSKCC scores would benefit from investigation in a larger cohort, and the good performance of IMDC relative to the MSKCC score aligns with previous work [3]. To our knowledge, the mccRCC cohorts analysed here are the largest available with RPPA data from pathologist-guided, multiregion tumour sampling. Our approach to capture grade diversity is likely to better represent ITH than standard sampling methods. Furthermore, each sample analysed by RPPA reflects a large tissue volume (*circa* 50–75 mm^3^) relative to standard approaches based on tissue sections from formalin-fixed paraffin embedded material such as tissue microarray analysis (<0.2 mm^3^ per region). Therefore, the RPPA data analysed cover a higher proportion of the overall tumour volume relative to standard approaches. The sampling approaches may be an important enabling factor in NEAT reproducibility and hence good validation performance, despite the relatively small cohorts studied. The RPPA technique offers potential as a quantitative alternative to IHC and has already been applied in a clinical setting through the Clinical Laboratory Improvement Amendments (CLIA) facility certification process [36, 37]. The NEAT model might ultimately be applied to inform decision making and patient management in several areas: (1) monitoring and follow-up, (2) recruitment into clinical trials with new agents, (3) treatment decisions, for example, for patients on the borderline of receiving drug due to other factors and (4) patient counselling.

The NEAT development and validation cohorts were relatively small (*n* = 44 total), which is associated with increased risk of type II error and wide confidence intervals on predictive performance. Cytoreductive nephrectomy is standard clinical practice, and the use of upfront tyrosine kinase inhibitor (TKI) treatment is variable, limiting recruitment of a uniform cohort (as was obtained from the SuMR clinical trial) for NEAT development. A further limiting factor on the size of cohorts in our study was the availability of appropriately consented fresh-frozen material with multiregion sampling and pathology assessment for RPPA analysis. Our approach to discover resistance biomarkers required multiregion sampling of tumour tissue from patients treated with upfront sunitinib in order to enable comparison of candidate marker variance in sunitinib-exposed and sunitinib-naïve material. Therefore, the cohorts received different treatment regimens and also had significant differences in some clinical characteristics. NEAT performed well on both cohorts despite these differences, and so might be broadly useful for prognostication of mccRCC. Further study of NEAT performance on an independent upfront sunitinib cohort would be of interest to further explore potential clinical utility, such as to inform decision making about performing a cytoreductive nephrectomy [38].

Subsampling of the multiregion RPPA data showed that validation of the NEAT prognostic model was critically dependent on the number of samples analysed per tumour. Indeed, the model's performance in risk stratification improved significantly at each increase in the number of tumour regions analysed (Fig. 5a). These results therefore evidence the benefit of more extensive tumour sampling both for biomarker development and also in validation studies where the sampling protocol may contribute to a reported lack of reproducibility. The efficacy of even the most promising tissue-based biomarkers is diminished by ITH [39], and identification of molecular predictors that are unaffected by ITH may be very challenging. Indeed, cancer biomarkers have historically suffered from a high attrition rate [8]. The available data provided for subsampling analysis of one, two and three samples per tumour; however, analysis with the full dataset (median of four samples) performed best. In principle, even higher sampling rates may be beneficial; several patients where >3 samples were taken, reflecting larger tumours, show considerable variation in HR even when large numbers of samples are analysed (Fig. 5b). One patient where eight tumour regions were examined had substantial variation in NEAT HR even across subsets containing six samples. Therefore, the influence of tumour sampling on predicted risk is clear for individual patients. These results also evidence benefit of sampling in proportion to tumour volume for molecular diagnostics. We found considerably greater variance in HR for low grade over high grade samples; thus, tumour biomarker studies would benefit from performing more extensive sampling of low grade regions. This result also underlines the additional information provided by NEAT. Indeed, the automatic feature selection process deprioritised grade relative to molecular variables. Prognostication using all of the multiple tumour samples gave better risk stratification than provided by analysis of any single sample in isolation. Therefore, NEAT analysis with multiple tumour regions captures information unavailable in any single sample; this information may reflect the adaptive potential arising from ITH [40] and also might include aspects of disease progression such as the degree of vascularisation or the length of time since initial dissemination competence.

With regard to the individual components of the NEAT model, the positive association of mTOR with overall survival was the strongest, most significant feature and was also found in univariate analysis of an overlapping cohort. The mTOR pathway is an important mediator of RTK growth signalling [41]. Improved prognosis associated with elevated mTOR in NEAT suggests that tumours dependent upon mTOR have enhanced sensitivity to sunitinib. Therefore, sunitinib may act directly on tumour cells to inhibit mccRCC growth, consistent with results in ovarian cancer that VEGF stimulates the mTOR pathway [42]. Additionally, the mTORC1 complex, which includes mTOR, exerts negative feedback on RTKs to suppress proliferation and survival [41]; this negative feedback could enhance therapeutic RTK inhibition by sunitinib. Notably, mTOR inhibitors are currently in clinical use (for example, everolimus), possibly in conjunction with sunitinib or similar agents. Our results suggest caution in co-treating with mTOR inhibitors and sunitinib, resonating with the poor performance of everolimus followed by sunitinib in the RECORD-3 trial [43]. Consistent with previous results, for example [44, 45], a significant negative association with survival was identified for N-cadherin, a canonical marker of epithelial to mesenchymal transition. Additionally, N-cadherin is expressed by endothelial cells and so may also represent a surrogate for vascularisation [46]. Age is a known RCC prognostic factor that was not selected for the IMDC score [3, 47, 48]. Our analysis took age as continuous values, which may partly explain selection of this variable for the NEAT model and not in the IMDC analysis, which dichotomised age at 60 years [49]. The IMDC score was not selected by our machine learning approach which implies that, in the development cohort, prognostic information captured by the IMDC score overlaps with that provided by the NEAT variables. High EPCAM expression is also associated with poor prognosis in NEAT and multiple cancers [50, 51], although reports link EPCAM with better prognosis in localised RCC; see, for example, [52, 53]. The contrasting association with survival for EPCAM in NEAT may be due to differences between advanced and localised ccRCC, technologies used and context-specific function, for example, in signal transduction by nuclear localisation of the cleaved intracellular domain [54].

## Conclusions

Multiregion sampling to capture mccRCC grade diversity enabled investigation of ITH impact on risk stratification with a novel protein-based prognostic model, NEAT (N-Cadherin, EPCAM, Age, mTOR). NEAT compares well with established clinico-pathological scores on a geographically separate independent validation cohort who received current standard therapy. Results show that evaluation or attempted use of any molecular prognostic and predictive methods with few tumour samples will lead to variable performance and low reproducibility. We demonstrate parameters (tumour coverage, size, grade) that may be used to inform sampling in order to enhance biomarker reproducibility, and results underline the critical importance of addressing heterogeneity to realise the promise of molecular stratification approaches. Through studies such as TRACERx [55], we anticipate that extensive multiregion sampling will become standard procedure for discovery and validation of molecular diagnostics across a range of cancer types.

Recommendations arising from our research include the following: (1) biomarker validation studies should implement tumour sampling protocols that match as closely as possible to the discovery work; (2) clinical biomarker research and ultimately front-line diagnostic approaches may benefit from greater tumour sampling rates; (3) clinical parameters (including tumour grade, size, coverage) can guide sample selection, and investigation of additional parameters to inform sampling may be useful; (4) optimisation of tumour sampling rate and sample selection protocols are important research areas to enable advances in stratified cancer medicine.

## Additional files

Additional file 1: Supplementary methods, tables and summary of the Additional file 2 zip archive (PDF). (PDF 61 kb)

Additional file 2: NEAT model, custom computer code and relevant datasets for investigation of limiting sample number on NEAT performance (ZIP archive). (ZIP 13 kb)

Additional file 3: Reverse phase protein array data for multiregion tumour sampling (comma-separated values, *CSV*). (CSV 141 kb)

Additional file 4: Clinical data; columns are: anonymised patient identifier, age, grade, event, overall survival, Heng class, MSKCC class. Anonymised identifiers correspond with those given in Additional file 3 (comma-separated values, *CSV*). (CSV 962 bytes)

## Abbreviations

BIC: Bayesian information criterion
ccRCC: Clear cell renal cell cancer
HR: Hazard ratio
IMDC: International Metastatic Database Consortium
ITH: Intratumoural heterogeneity
mccRCC: Metastatic clear cell renal cell cancer
MNTS: Maximum number of tumour samples
mOS: Median overall survival
MSKCC: Memorial Sloan Kettering Cancer Center
NEAT: N-cadherin EPCAM Age mTOR multivariate model
OS: Overall survival
RCC: Renal cell cancer
RPPA: Reverse phase protein array
RTK: Receptor tyrosine kinase
SCOTRRCC: Scottish Collaboration On Translational Research into Renal Cell Cancer
