# Supplementary material for: Overcoming intratumoural heterogeneity for reproducible molecular risk stratification: a case study in advanced kidney cancer
Source: BMC Med. 2017 Jun 26;15:118. doi: 10.1186/s12916-017-0874-9 (PMC5483837; doi:10.1186/s12916-017-0874-9)
Supplement: Supplementary file 1 — Supplementary methods, tables and summary of the Additional file 2 zip archive (PDF). (PDF 61 kb) [file 12916_2017_874_MOESM1_ESM.pdf]

# Supplementary Material for “Overcoming Intratumoural Heterogeneity for Reproducible Molecular Risk Stratification: A Case Study in Advanced Kidney Cancer”

**CONTENTS**


| | Page |
|---|---|
| Supplementary Methods ........................................................................... | 2 |
| Supplementary Table S1 ........................................................................... | 3 |
| Supplementary Table S2 ........................................................................... | 3 |
| Overview of Additional File 2 Zip Archive ........................................................ | 4 |

**Supplementary Methods**

*Tumour subsampling procedure and statistical comparisons*

For the values of maximum number of tumour samples (MNTS) analysed, there were between 2.35x$10^8$ and 2.74x$10^{11}$ possible tumour sample combinations across the validation cohort (2.35×$10^8$ for MNTS=3; 2.74×$10^{11}$ for MNTS=2; 1.16×$10^{11}$ for MNTS=1). Our analysis took one million combinations for each MNTS using Sobol sampling, a quasi-random, low discrepancy approach to ensure uniform sampling [29].

The number of possible tumour sample combinations for each MNTS is given by the product of the number of sample combinations per patient: $N_i$ choose$\{k\}$, where $N_i$ indicates the number of samples available for patient i (2-8 for the validation cohort) and $\{k\}$ is the specified MNTS. For patients with number of samples fewer than or equal to the specified MNTS, all samples were used. Each of the possible sample combinations across the cohort for a given MNTS may be indexed by a unique integer; one million such integers were generated using a Sobol sequence [29]. The mapping of each (master) integer generated by the sequence to a list of samples for each patient was calculated in two steps. Firstly, the master integer was converted to a set of integers, one per patient, which represented the combinadic (combinatorial index) for each patient's sample combination. This was done by converting the master integer to a mixed radix number, where a radix equal to $N_i$ choose$\{1,2,3\}$ was present for each patient, which is interpreted as a patient's sample combinadic. Subsequently, each combinadic was mapped onto the specific samples for the corresponding patient for inclusion in the sampling run. Distributions of HR for each MNTS were compared using the Mann-Whitney test. The comparison to random sampling for the MNTS=1 HR using the binomial test took an even distribution of samples either side of logHR=0 as the null distribution (representing no prognostic information), hence a probability of success=0.5; the NEAT logHR distribution for MNTS=1 was tested for a shift towards values >0.

**Supplementary Table S1. Antibodies used for candidate molecular variables.**

| **Antibody** | **Supplier** |
|---|---|
| mTOR | Cell Signalling (2972) |
| EPCAM | Cell Signalling (2929) |
| N-Cadherin | BD (610921) |
| BCL2 | Eurogentech (75380) |
| MLH1 | Cell Signalling (3515) |
| CA9 | Novus Biologicals (NB 100-417) |

**Supplementary Table S2. Results of Grambsch-Therneau test of proportional hazards assumption for NEAT model**

| | rho | chisq | *p* |
|---|---|---|---|
| mTOR | -0.245 | 0.929 | 0.335 |
| EPCAM | 0.12 | 0.162 | 0.687 |
| N_Cad | 0.15 | 0.235 | 0.628 |
| Age | 0.194 | 0.653 | 0.419 |
| GLOBAL | n/a | 1.728 | 0.786 |

**Summary of Additional File 2 Zip Archive**

The zip archive 'AdditionalFile2.zip' provides metadata, data and computer code used to study the effects of intratumoural sampling on the NEAT prognostic model validation performance. The following files are included:

*README.md* – Metadata, including important information about the computer code.

*NEAT-model.RData* - An RData object containing the NEAT model ("coxph" class object from R's "survival" library)

*combinadics.R* - R functions for combinadic and mixed radix arithmetic

*dosampling.R* - R script for performing the sampling procedure detailed in Supplementary Methods and in the README.md file

*expressiondata.csv* – Data file providing anonymised per-sample RPPA expression data for the three NEAT protein markers (N-Cadherin, EPCAM and mTOR)

*patientdata.csv* – Data file with anonymised ages, survival time and survival status

*LICENSE.txt* – The Creative Commons CC-BY-NC-SA license
